# IL-Y Aggravates Murine Chronic Graft-*Versus*-Host Disease by Enhancing T and B Cell Responses

**DOI:** 10.3389/fimmu.2020.559740

**Published:** 2020-11-23

**Authors:** Li Wan, Ziqi Jin, Bo Hu, Kangkang Lv, Lei Lei, Yonghao Liu, Yuan Song, Ying Zhu, Huanle Gong, Mimi Xu, Yuanyuan Du, Yang Xu, Haiyan Liu, Depei Wu, Yuejun Liu

**Affiliations:** ^1^ National Clinical Research Center for Hematologic Diseases, Jiangsu Institute of Hematology, The First Affiliated Hospital of Soochow University, Institute of Blood and Marrow Transplantation, Suzhou, China; ^2^ Collaborative Innovation Center of Hematology, Soochow University, Suzhou, China; ^3^ Immunology Programme, Life Sciences Institute and Department of Microbiology and Immunology, Yoo Loo Lin School of Medicine, National University of Singapore, Singapore, Singapore

**Keywords:** IL-Y, T cell response, B cell response, Tfh cell, Treg cells, chronic graft-*versus*-host disease

## Abstract

IL-Y, a synthetic member of IL-12 cytokine family, was found to exert potent immunosuppressive effects by inhibiting the differentiation and activation of Th1 and Th17 cells. However, the role of IL-Y in the development of chronic graft-*versus*-host disease (cGVHD) remains unknown. Here, using murine models of scleroderma-like and lupus-like cGVHD, we examined the function of IL-Y in the pathogenesis of cGVHD by hydrodynamically injecting minicircle-IL-Y expressing plasmids (MC IL-Y). In contrast with the reported immune suppressive function of IL-Y, administration of MC IL-Y enhanced cGVHD severity reflected by deteriorated multi-organ pathologic damages. In lupus-like cGVHD model, urine protein and the serum anti-dsDNA antibody (IgG) were significantly upregulated by IL-Y treatment. Further study demonstrated that IL-Y impacts both donor T and B cell response. In T cells, IL-Y inhibited the generation of CD4^+^Foxp3^+^ regulator T (Treg) cells during the development of cGVHD. IL-Y may also increase the infiltration of pathogenic TNF-α producing CD4^+^ and CD8^+^ T cells through IL-27R*α* in recipient spleens, as this effect was diminished in IL-27R*α* deficient T cells. Moreover, IL-Y enhanced the differentiation of ICOS^+^ T follicular helper (Tfh) cells. In B cells, the percentage of germinal center (GC) B cells in recipient spleens was significantly upregulated by MC IL-Y plasmid administration. The levels of co-stimulatory molecules, MHC-II and CD86, on B cells were also enhanced by IL-Y expression. Taken together, our data indicated that IL-Y promoted the process of cGVHD by activating pathogenic T and B cells.

## Introduction

Allogeneic hematopoietic stem cell transplantation (Allo-HSCT) remains a cornerstone curative therapy for hematological malignancy. Chronic graft-*versus*-host disease (cGVHD) continues to be a common cause of non-relapse morbidity and mortality after allo-HSCT (1–4). With standard prophylaxis based on a calcineurin inhibitor and methotrexate or mycophenolate mofetil in most regimens, fewer patients have developed acute GVHD (aGVHD) in recent years. However, the incidence and clinical therapy of cGVHD have not been improved due to the poor understanding of its pathogenesis. Paradoxically, cGVHD prophylaxis and treatment with a calcineurin inhibitor may promote the development of cGVHD by blocking thymic central tolerance and peripheral Treg-cell function (5, 6). More seriously, it is closely associated with increased risk of infection and malignancy recurrence (2, 7). Therefore, new therapeutic strategies are urgently needed to improve curative effect of cGVHD.

cGVHD is a multi-system autoimmune-like syndrome caused by the interactions of donor T and B cells and antibody production, with clinical manifestations including skin and cutaneous sclerosis, bronchiolitis obliterans as well as salivary and lacrimal gland pathology (2–4, 8). The pathogenic auto-reactive and allo-reactive CD4^+^ T cells escape immune regulation by thymic selection and peripheral mechanisms and differentiate into type 1, type 2, and type 17 helper T (Th1, Th2, and Th17) cells, which maintain inflammation (9–12). It is evident that donor B cells also contribute to the immune pathology and tissue damage characteristic of cGVHD (4, 8, 12, 13). Activated follicular helper T cells expressing the transcription factor Bcl6 and high levels of the chemokine receptor CXCR5 support the generation of germinal center (GC) B cells by providing signaling through IL-21, ICOS, and CXCL13 (11, 14, 15). Interaction of T follicular helper (Tfh) and B cells results in somatic hypermutation, production of high afﬁnity IgG, and formation of long-lived plasma cells, which exacerbate the development of cGVHD (11, 12, 16). Regulatory T (Treg) cells, follicular regulatory T (Tfr) cells, regulatory B (Breg) cells represent peripheral immune tolerance, which also plays a critical regulatory role in the pathogenesis of cGVHD (14, 17–20).

Previous studies using western blot followed by immune-precipitation revealed that a stable association between p28 and p40 was formed possibly *via* disulfide bond (21). Injection of p28/p40 protein suppressed experimental autoimmune uveitis by inhibiting the differentiation and inflammatory responses of Th1 and Th17 cells. These suppressive effects seemed to be ascribed to antagonizing the activation of STAT1 and STAT3 pathways induced by IL-27 and IL-6, both of which signal through the gp130 receptor (21). Moreover, recent studies using adenovirus vector expressing p28/p40 (IL-Y) suggested that treatment of pre-diabetic non-obese mice prevented the onset of hyperglycemia with reduced expression of inflammatory mediators such as IFN-*γ* (22). Interestingly, their work also demonstrated that IL-Y could activate antigen-presenting cells (APCs) by significantly upregulating both CD86 and MHC-II expression on myeloid derived-suppressor cells (MDSCs) (22). Therefore, these studies implicated that IL-Y might play a dual role in immune regulation.

Given that cGVHD has a wide spectrum of presentations in humans, individual mouse models do not reproduce all features of cGVHD. We investigated how IL-Y regulated T and B cells differentiation and function during cGVHD development in two mouse models of cGVHD, scleroderma-like cGVHD model and lupus-like cGVHD model. We observed that IL-Y aggravated the development of autoimmune manifestations of cGVHD. Furthermore, we found that IL-Y administration increased ICOS^+^ Tfh cells, promoted the production of TNF-α, inhibited Treg generation, and enhanced the differentiation of B cells to GC B cell. Although the detailed mechanisms of IL-Y promoting cGVHD require further exploration, our results provide a new insight in the role of IL-Y in cGVHD and possible therapeutic strategies targeting p40 (a component of IL-Y) and IL-27R*α* signaling.

## Materials and Methods

### Mice

8–10-week-old female DBA/2 (H2K^d^) mice were purchased from Charles River Laboratories (Beijing, China). 6–8-week-old female C57BL/6 (B6; H2K^b^) and BALB/c (H2K^d^) mice were purchased from SLAC Animal Laboratory (Shanghai, China). Experimental animals were maintained in specific pathogen-free conditions. All animal protocols were approved by the Soochow University Institutional Animal Care and Use Committee.

### Establishment of cGVHD in BALB/c Mice

Recipient BALB/c mice were conditioned with total body irradiation (TBI) at 650 cGy using an RAD 320 X-ray Irradiator 6–8 h prior to transplant. Irradiated recipients (BALB/c) were intravenously injected with 1 × 10^7^ bone marrow (BM) cells and 1 × 10^6^ whole splenocytes (C57BL/6J→BALB/c) to establish scleroderma-like cGVHD model. 5 × 10^6^ BM cells and 4 × 10^7^ CD4^+^CD25^−^ splenocytes were injected intravenously to irradiated recipients (BALB/c) (DBA/2→BALB/c) to establish lupus-like cGVHD model. CD25 depletion in the spleens was accomplished using biotin-conjugated anti-CD25 mAb (eBioscience, San Diego, California) and anti-biotin micromagnetic beads (Miltenyi Biotec, German), followed by passage through a MACS cell sorter (Miltenyi Biotec, German). The efficiency of depletion was >98%. For hydrodynamic gene transfer (HGT), the recipient mice (BALB/c) were injected intravenously with 120 µg of empty vectors (MC) or minicircle-IL-Y (MC IL-Y) plasmids in a total of 2 ml phosphate buffered saline (PBS) within 5 s using a 23-gauge needle 3 days before transplantation.

### Plasmid Construction

The cDNA encoding mouse IL-27p28 and IL-12p40 were amplified by PCR from the total RNA extracted from spleen cells of C57BL/6 mice stimulated with LPS. IL-27p28 and IL-12p40 genes were fused *via* a hydrophobic polypeptide linker (Gly4Ser). The IL-Y expression construct was generated by fusing the nucleotide sequence-encoding Igκ signal sequence to the 5′ end of IL-Y sequence and flag tag to the 3′ end of IL-Y sequence, and then inserted between sites of Nhe I (5′) and Sal I (3′) into minicircle (MC) plasmid (pMC.EF1; SBI, Palo Alto, CA). Positive recombinant clone was analyzed by digestion of restriction endonuclease and DNA sequencing.

### Serum Anti-dsDNA Antibody Detection

We made double-stranded DNA (dsDNA) from calf thymus (Sigma, D1501). High-binding ELISA plates (Costar, 3369) were coated with a mixture containing 50 µg/ml dsDNA 2 h at 37°C and then incubated at 4°C overnight. The plates were then blocked with NaCO_3_/NaHCO_3_ buffer solution containing 5% goat serum for 1 h at 37°C. Following blocking, plates were washed several times with 0.05% tween-20 PBS (PBST). Serum samples were added at 1:100 ratio in PBST containing 10% new bovine serum (NBS) and 5% goat serum. Plates were incubated at 37°C for more than 2 h and then washed with PBST for three times. The HRP-conjugated secondary antibody (HRP-IgG or HRP-IgG1 or HRP-IgG2a) (Southern Biotech, Birmingham, Alabama) was then added at a 1:1,000 ratio in PBST containing 10% NBS and 5% goat serum and incubated for 1 h at 37°C. Plates were then washed four times, and 50 µl of TMB Substrate (eBioscience, San Diego, CA) was added to each well. After 15–30 min, the reaction was stopped using 50 µl of 1 mol phosphoric acid, and the plate was read at 450 nm. Wells with no serum were used as negative controls. Plates were read by a SYNERGY-HTX ELISA plate reader (BioTek, Vermont).

### Assessment of cGVHD and Histopathology

Recipient mice were monitored for survival, weight loss, and clinical scores of cGVHD. Urine protein was detected by BCA Protein Assay Kit (Beyotime Biotechnology, China). In order to quantify the histopathologic parameters of GVHD target organs, salivary, kidney, skin, lung, liver, thymus, and small intestine of recipient mice were collected 56 days post BM transplantation. Tissues were fixed with 10% formalin and made into slices with hematoxylin and eosin (HE) staining and observed under optical microscope (Nikon, Japan). Tissue damage was blindly assessed on a scoring system described previously (23). In particular, a numeric value was attributed to the changes observed in the kidney (loss of glomeruli, architecture disruption, immune complex deposition, lymphocytes infiltration), in the skin (dermal fibrosis, fat loss, epidermal thickening, follicular loss, and inflammation), in the lung (perivascular and peribronchiolar infiltration, pneumonitis alveolar/interstitial), in the liver (number of involved tracts, lymphocytic infiltration, liver cell necrosis), in the small intestine (mucosal, lamina propria, muscular, serosal). Collagen deposition was quantified by measuring percent of blue area in ImageJ.

### Flow Cytometry

Single cell suspensions were obtained according to the methods previously described and stained for surface receptors and intracellular cytokines. The antibodies and reagents used for flow cytometry analysis were listed as below: antibodies to mouse, PE-CF594-CD3e (145-2C11), PE/Cy7-B220 (RA3-6B2), Allophycocyanin-CD138 (281-2), BV421-Gl-7 (Gl-7), rat BV421-IgG2a isotype control (R35-95) BV510-CD95 (Fas, Jo2), PE-CD278 (ICOS, 7E.17G9), PE-IL-27R*α* (2918), PE-IL-12R*β*1 (3C9) were purchased from BD Bioscience (San Diego, CA); Allophycocyanin-CD185 (CXCR5, L138D7), Pacific Blue-CD8 (Gl-1), Allophycocyanin/Cy7-IL-17A (TC11-18H10.1), PE-CD21 (7E9), PE/Cy7-CD44 (IM7), PE-IFN-γ (XMG1.2), Allophycocyanin/Cy7-CD23 (B3B4), PE/Cy7-TNF-α (MP6-XT22), PE-Foxp3 (MF-14) Allophycocyanin-IL-4 (11B11), purified anti-mouse IL-12/IL-23 p40 and purified CD16/32 were purchased from Biolegend (San Diego, CA). Foxp3 staining kit was purchased from eBioscience (San Diego, CA). Flow cytometric analysis was performed using a FACS NovoCyte (ACEA Biosciences, San Diego, CA) and the Flowjo software (Tree Star, Ashland, OR).

### Serum Cytokine Analysis

The levels of IL-2, IL-4, IL-17A, IFN-*γ*, TNF-α and IL-21 in serum were quantiﬁed by Cytometric Beads Array (CBA) kit (BD Bioscience, San Diego, CA).

### T Cell Activation Assay

Naive T cells were sorted from splenocytes of C57BL/6 mice by Mouse Pan-Naive T Cell Isolation Kit according to the manufacturer’s protocol (StemCell Technologies, Vancouver, Canada). Plates were coated with 1 μg/ml anti-CD3 and 0.2 μg/ml anti-CD28 Abs (BioLegend) overnight. A total of 1 × 10^5^ naive T cells were cultured for 48 h alone or with 10 μg/ml rIL-Y protein (DETAIBIO, China). T cells were then analyzed by flow cytometry to determine the TNF-α production of CD4^+^ T cells and CD8^+^ T cells.

### Statistics

Statistical analyses and data presentation were performed using GraphPad Prism 5 software for Mac (Graphpad Software, San Diego, CA). Unpaired Student tests were used to determine statistically significant differences between two experimental groups. Data are expressed as mean ± SD. P value < 0.05 was considered statistically significant (*), less than 0.01 or 0.001 was shown as ** or ***, respectively.

## Results

### IL-Y Promotes the Development of Murine Lupus-Like and Scleroderma-Like cGVHD

**Figure 1:**
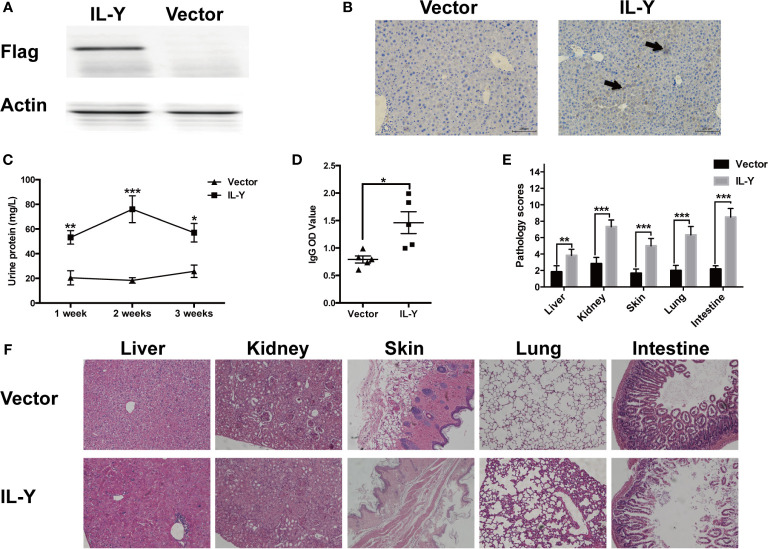
IL-Y promotes the development of murine lupus-like cGVHD. **(A, B)** Mice were hydrodynamically injected with empty plasmids or IL-Y MC plasmids. IL-Y expression was detected by western blot **(A)** and immunohistochemistry **(B)** (original magnification ×40) in liver 7 days after plasmid injection by HGT. BALB/c mice were lethally irradiated (6.5 Gy) and transferred with 5 × 10^6^ BM cells and 4 × 10^7^ CD4^+^CD25^−^ splenocytes 3 days after plasmid injection by HGT. **(C)** Urine protein was detected by BCA Protein Assay Kit. **(D)** Serum level of anti-dsDNA IgG was determined. **(E)** Pathology scores are shown 8 weeks after donor cell transfer. **(F)** Representative histopathology photos of liver, kidney, skin, lung, and intestine are shown. Data are representatives of at least three independent experiments. Values are presented as means ± SD. *P < 0.05; **P < 0.01; ***P < 0.001.

Murine IL-Y expression construct was generated by fusing IL-27p28 and IL-12p40 *via* a hydrophobic polypeptide linker (Gly4Ser). The nucleotide sequence-encoding Igκ signal sequence was fused to the 5′ end of IL-Y sequence and flag tag was inserted to the 3′ end of IL-Y sequence, then full-length IL-Y was inserted between sites of Nhe I (5′) and Sal I (3′) into minicircle (MC) plasmid. IL-Y release in the liver was achieved by hydrodynamically injecting MC IL-Y plasmids. IL-Y expression in the liver was confirmed by immunohistochemistry and western blot 7 d after plasmid injection (**Figures 1A, B** **)**.

**Figure 2:**
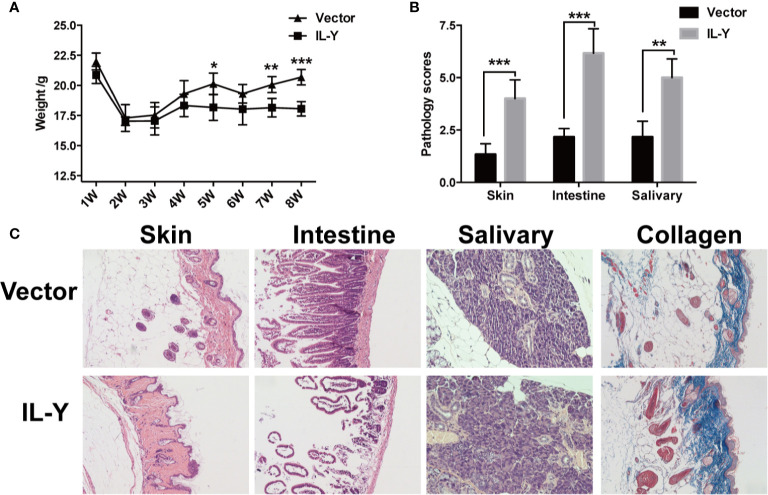
IL-Y promotes the development of murine scleroderma-like cGVHD. BALB/c (H2K^d^) mice (n = 6 each group) were lethally irradiated and transferred with 1 × 10^7^ BM cells and 1 × 10^6^ splenocytes of C57BL/6 (H2K^b^) mice 3 days after plasmid injection by HGT. **(A)** Mice body weight is shown. **(B)** Pathology scores are shown 8 weeks after donor cell transfer. **(C)** Representative histopathological pictures of skin, intestine, and salivary gland, as well as Masson’s trichrome staining are shown. Data are representative of at least three independent experiments. Values are presented as means ± SD. *P < 0.05; **P < 0.01; ***P < 0.001.

To examine the role of IL-Y in the development of cGVHD in mice, we established a lupus-like cGVHD model that is featured with autoimmune manifestations including autoantibody production, glomerulonephritis, proteinuria and ascites. IL-Y MC plasmids were hydrodynamically injected 3 days before irradiation. Bone marrow cells and CD4^+^CD25^−^ splenocytes isolated from DBA/2 mice were injected intravenously into lethally irradiated (6.5 Gy) Balb/c recipients. IL-Y significantly increased the level of urine protein at 1 week, 2 weeks and 3 weeks post bone marrow transplantation (BMT) (**Figure 1C**). In addition, mice administrated with MC IL-Y plasmids displayed significantly higher level of serum IgG autoantibodies (**Figure 1D**). Histologic assessment revealed more severe tissue damage in the liver, kidney, skin, lung, as well as small intestine in recipients with IL-Y MC plasmids administration (**Figures 1E, F** **)**. To further exclude the model specific phenomenon, we established scleroderma-like cGVHD model to confirm the pathogenic role of IL-Y in the development of cGVHD. Specifically, Balb/c mice were injected with 1 × 10^6^ spleen cells and 1 × 10^7^ bone marrow cells from C57BL/6 mice after irradiation (6.5 Gy) 3 days after IL-Y MC plasmids injection. In the late stages of cGVHD, mice in the MC IL-Y group showed more weight loss (**Figure 2A**). Consistently, IL-Y also significantly aggravated the histopathology damage compared with empty vector control in this scleroderma-like cGVHD model (**Figures 2B, C** **)**. As sclerosis is an important feature of cutaneous cGVHD, collagen deposition was found to be significantly increased in the recipient skin in MC IL-Y plasmids group (**Figure 2C**). Altogether, these results indicated that IL-Y promoted the development of cGVHD.

### IL-Y Suppresses Treg Differentiation and Facilitates Tfh Activation

**Figure 3:**
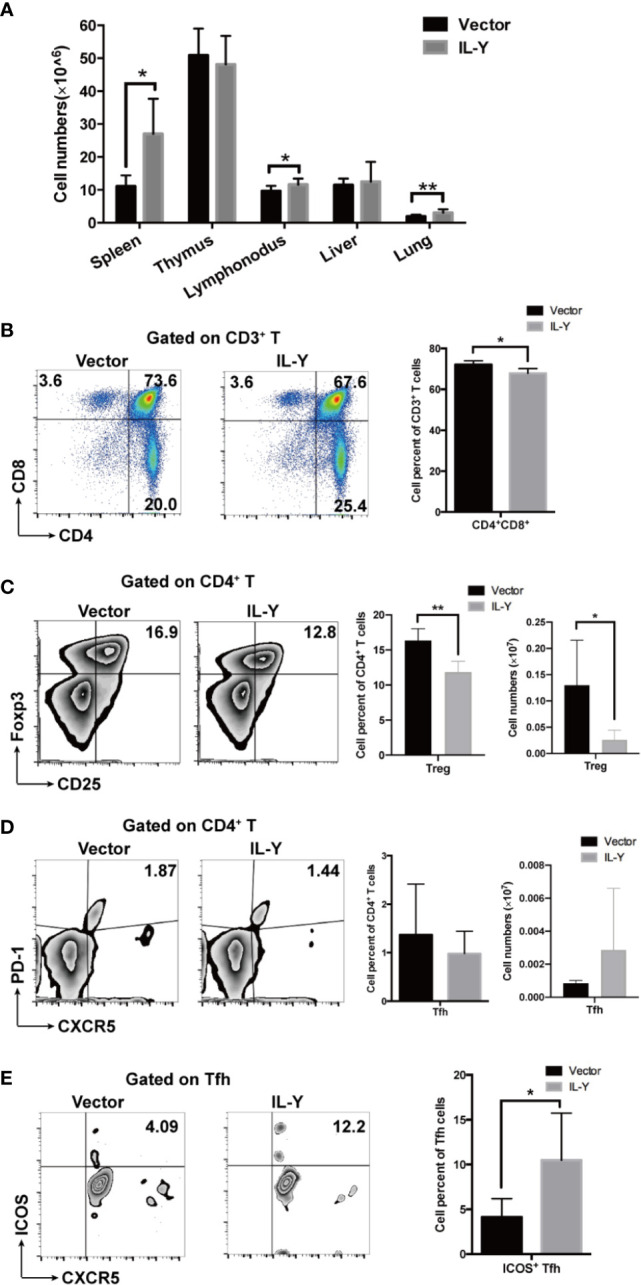
IL-Y suppresses Treg differentiation and facilitates Tfh activation. Splenocytes (n = 6 each group) were collected and stained for FACS analysis 8 weeks after donor cell transfer. **(A)** Numbers of lymphocytes infiltration in different organs are shown. **(B)** Percentage of CD4^+^ CD8^+^ T cells in thymus is shown. **(C)** Percentage and number of CD4^+^ CD25^+^ Foxp3^+^ Treg cells are shown. **(D)** Percentage and number of Tfh cells (CD4^+^ PD-1^+^ CXCR5^+^) are shown. **(E)** Percentage of ICOS^+^ Tfh cells is shown. Data are representatives of at least three independent experiments. Values are presented as means ± SD. *P < 0.05; **P < 0.01; *P* < 0.001.

To further explore the underlying mechanism how IL-Y exacerbates the development of cGVHD, we examined the splenic immune cell phenotypes 8 weeks after lupus-like cGVHD model establishment. Lymphocytes infiltration was significantly increased in spleens, lymph nodes and lungs in IL-Y group (**Figure 3A**). As a direct target organ, thymus is heavily involved in cGVHD pathogenesis by inducing auto-reactive emigrants and impairing Treg generation (12). Consistent with the aggravated clinical manifestations, MC IL-Y plasmids treated mice displayed significantly lower percentage of CD4^+^ CD8^+^ T cells in thymus (**Figure 3B**). Previous studies pointed to pro-inflammatory cytokines produced by pathogenic CD4^+^ T cells, Th1 and Th17 cells, as the driving force for the initiation of cGVHD (11, 17, 24). In addition, donor CD8^+^ T cells preferentially damaged recipient medullary thymic epithelial cells and impaired negative selection, resulting in production of auto-reactive CD4^+^ T cells that perpetuated the damage to the thymus and augmented the development of cGVHD (10). However, we did not observe significant increase of activated and effector CD4^+^ and CD8^+^ T cells in MC IL-Y group (data not shown).

Treg cells play an important role in maintaining immune-tolerance, preventing autoimmune diseases and limiting inflammatory diseases, including cGVHD. Treg population at early time points is crucial for modulating GVHD. In our cGVHD model, percentage and number of Treg cells were significantly decreased in MC IL-Y plasmids group 2 weeks after cGVHD model establishment (**Figure 3C**). At 8 weeks post transplantation, the percentage of Treg cells was also decreased in the IL-Y group (**Figure S1A**). Studies have shown that Tfh cells also play an extremely important role in the pathogenesis of cGVHD by promoting GC B cell formation and B cell activation (12, 15, 16, 25). Interestingly, patients and murine model with active cGVHD have decreased numbers of Tfh cells compared with no or mild cGVHD, but Tfh cells expressed high levels of ICOS and secreted higher levels of CXCL13 in plasma to facilitate contact between Tfh and B cells (11, 16). Indeed, we found that there was no difference in the percentage and number of Tfh cells in spleens (**Figure 3D**). However, the percentage of ICOS^+^ Tfh cells was significantly increased in spleens by IL-Y expression (**Figure 3E**), which indicated that IL-Y might promote GC formation and B cell activation *via* promoting ICOS^+^ Tfh differentiation. T follicular Regulatory (Tfr) cells restrain GC responses by inhibiting Tfh and B cell function (18, 20). However, there was no difference in the subgroup of Tfr cells (data not shown). These results suggested that IL-Y selectively inhibited Treg cell differentiation and promoted Tfh activation to facilitate the development of cGVHD.

### IL-Y Increases Germinal Center B Cell Responses and B Cell Function

**Figure 4:**
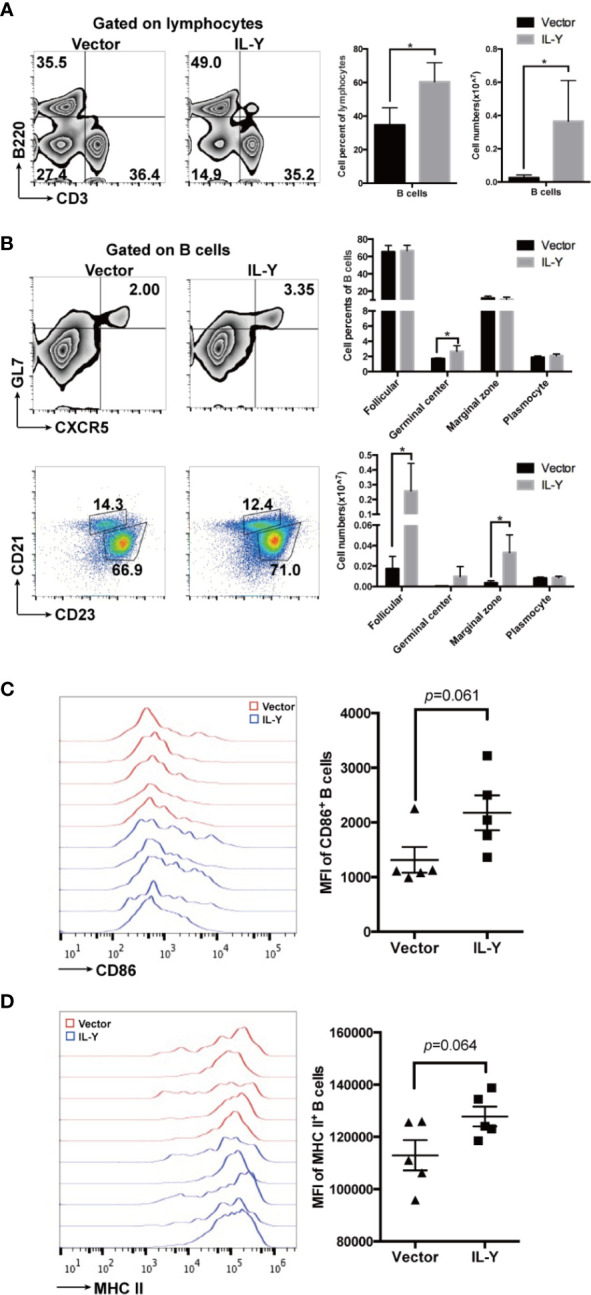
IL-Y increases germinal center B responses and B cell function. Splenocytes (n = 6 each group) were collected and stained for FACS analysis 8 weeks after donor cell transfer. **(A)** Percentage and number of splenic B cells (CD3^−^ B220^+^) are shown. **(B)** Percentage and number of follicular (CD21^+^ CD23^+^), marginal zone (CD21^hi^ CD23^low^), GL7^+^ CXCR5^+^ germinal center B cells and plasmocytes (CD138^+^) gated on CD3^−^ B220^+^ B cells are shown. Further analysis of B cell phenotypes. **(C, D)** Mean fluorescence intensity (MFI) of CD86 and MHC-II is shown. Data are representatives of at least three independent experiments. Values are presented as means ± SD. *P < 0.05.

B cells exacerbated the development of cGVHD through GC B cell formation, antibody production, and antigen presentation to T cells. Previous studies indicated that the administration of B cell-depleting anti-CD20 could ameliorate cGVHD in some patients (26, 27). We found that MC IL-Y plasmid treatment significantly increased both percentage and number of splenic B cells (**Figure 4A**). Further analysis of B cell phenotypes showed that there was increased percentage of GC B cells in MC IL-Y plasmids group (**Figure 4B**). In addition, the numbers of follicular B cells and marginal zone B cells were upregulated by IL-Y expression (**Figure 4B**). Levels of co-stimulatory molecules, including CD86 and MHC-II, were found upregulated on donor B cells in MC IL-Y plasmids group (**Figures 4C, D** **)**, suggesting that IL-Y may affect B cell activation and antigen presenting function. Taken together, these data indicated that IL-Y exacerbated lupus-like cGVHD by promoting B cells activation and function.

### IL-Y Promotes TNF-α Production by CD4^+^ and CD8^+^T Cells *In Vivo* and *Vitro*

**Figure 5:**
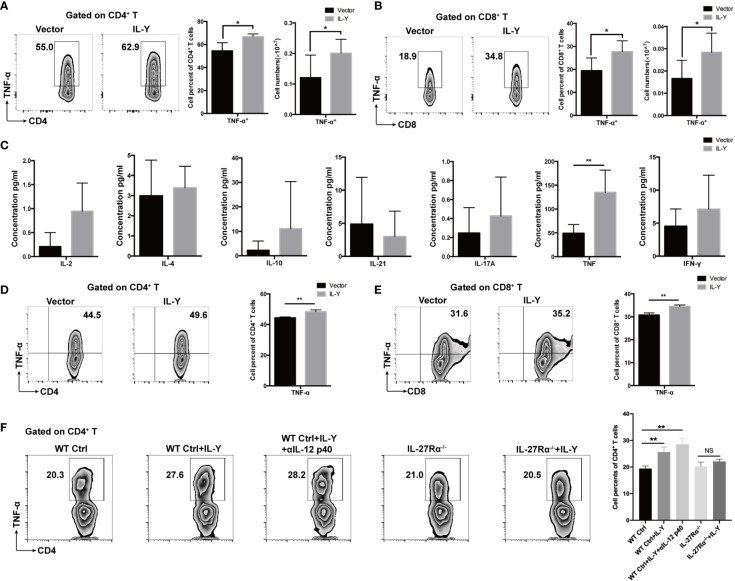
IL-Y promotes generation of TNF-α by CD4^+^ and CD8^+^ T cells *in vivo* and *in vitro*. ***(*A, B)** Splenocytes (n = 6 each group) were collected 8 weeks after donor cell transfer. Splenocytes and intrahepatic leukocytes were stimulated and then analyzed for intracellular cytokine production. Percentages and numbers of TNF-α-producing CD4^+^ T cells and CD8^+^ T cells in spleens are shown. **(C)** Serum levels of IL-2, IL-4, IL-10, IL-21, IL-17A, TNF-α, and IFN-*γ* were measured by CBA assays. **(D, E)** Naïve T cells were sorted from spleens of mice and cultured in plates coated with anti-CD3 and anti-CD28 antibodies and with 100 ng/ml rIL-Y or PBS for 48 h. Percentages of TNF-α-producing CD4^+^ T cells and CD8^+^ T cells are shown. **(F)** Naïve T cells from WT and IL-27R*α* ^−/−^ mice spleens were sorted and cultured in plates coated with anti-CD3 and anti-CD28 antibodies and with 100 ng/ml rIL-Y or PBS or 1 μg/ml anti-IL-12 p40 for 48 h. Percentage of TNF-α-producing CD4^+^ T cells is shown. Data are representatives of at least three independent experiments. Values are presented as means ± SD. NS indicates no significant difference. *P < 0.05; **P < 0.01.

We then examined the impacts of IL-Y on the shifting of cytokine balance in the splenocytes of recipients. Both percentage and number of TNF-α-producing CD4^+^ and CD8^+^ T cells were markedly elevated by IL-Y expression (**Figures 5A, B** **)**. Moreover, TNF-α secretion by CD4^+^ T cells and CD8^+^ T cells were also significantly upregulated in livers (data not shown). We did not observe significant changes in IL-17, IL-10 and IFN-*γ* (data not shown). Serum levels of IL-2, IL-4, IL-10, IL-17A, IFN-*γ*, TNF-α and IL-21 were determined by CBA assays. Consistently, serum TNF-α level was elevated by IL-Y expression (**Figure 5C**). To investigate whether IL-Y can directly promote TNF-α production by CD4^+^ and CD8^+^ T cell, we stimulated naive T cells from the spleen with anti-CD3/anti-CD28 *in vitro* and analyzed the TNF-α-producing CD4^+^ and CD8^+^ T cells subsets in the presence or absence of rIL-Y. The percentages of both TNF-α-producing CD4^+^ and CD8^+^ T cells were significantly increased (**Figures 5D, E** **)**. Flores et al. suggested IL-Y exerted its suppressive effect through IL-27R*α* (22); this effect was proved diminished in IL-27R*α* deficient mice (**Figure 5F**). In addition, the role of IL-12Rβ1 signaling cannot be ignored because IL-12/23 p40 was found to play critical roles in the development of GVHD. However, blockade of IL-12 p40 had no effect on the secretion of TNF-α by CD4^+^ T cells (**Figure 5F**). The role of IL-27R*α* signaling in GVHD is still not clear. It has been demonstrated that IL-27R*α* signaling on T cells deteriorates GVHD severity by promoting Th1 responses (28) and IL-27R*α* signaling blockade reduced GVHD (29), while Le et al. suggested that IL-27 stimulation enhanced Treg functions to prevent GVHD (30). Thus, differential expression of IL-27R*α* on different T cell subsets may paly contrary role during GVHD. We detected the expression of IL-27R*α* on CD3^+^ T cells and Treg cells 14 days post-transplant. The results showed no difference between the IL-Y and the control group (**Figures S1B, C**). Collectively, these data indicated that IL-Y could selectively promote TNF-α production by CD4^+^ and CD8^+^ T cells, which probably signals through IL-27R*α* and presumably contributed to the progression of cGVHD.

## Discussion

Chronic graft-*versus*-host disease (cGVHD) is a major complication in the late stage of allo-HSCT. With the decrease of mortality in the early stage of transplantation, the increase of the upper limit of the recipients’ age, the application of unrelated donors and peripheral blood hematopoietic stem cells, the incidence of cGVHD gradually increases, which adversely affects the life quality of allo-HSCT patients. cGVHD has become an important cause of non-recurrent death in the late stage of transplantation (7, 31). In the current study, we constructed a MC IL-Y eukaryotic expression plasmid and examined the pathogenic role of this novel cytokine in the development of cGVHD. We demonstrated that IL-Y played a critical role in the pathogenesis of cGVHD *via* activating T and B cell responses, and subsequent occurrence of scleroderma and antibody deposition in murine models of cGVHD. Mechanistically, IL-Y could accelerate the initiation of cGVHD by enhancing pro-inflammatory cytokine TNF-α production by pathogenic T cell. In addition, IL-Y perpetuates the pathogenesis of cGVHD by enhancing GC cell responses and antibody production.

Sakoda et al. found that self-reactive donor T cells played an important role in the development of cGVHD and improvement in the thymic function may have a potential to reduce cGVHD (32). Research of Wu et al. showed that donor CD8^+^ T cells preferentially damaged recipient medullary thymic epithelial cells and impaired negative selection, resulting in production of autoreactive CD4^+^ T cells, which perpetuated damage to the thymus and augmented the development of cGVHD (10). As a direct target organ, thymus was heavily involved in cGVHD pathogenesis, and its damage induces autoreactive emigrants and impairs Treg generation (10, 32). Consistent with the aggravated clinical manifestations observed, significant lower percentages and numbers of CD4^+^ CD8^+^ T cells in thymus were found in the recipients of IL-Y administrated mice. This further suggests that IL-Y may aggravate cGVHD by impairing thymus development.

It has traditionally been assumed that the predominant cytokines produced during cGVHD are Th2 cytokines, which can stimulate host B cell autoantibody production (33, 34). Recent studies have suggested that cGVHD could be caused by cytokines secreted by Th1 and Th17 cells (9, 11, 24). Previous prospective studies have found that levels of serum TNF-α in cGVHD patients were associated with disease severity (35). Similarly, high levels of TNF-α can be detected in patients with systemic sclerosis (36). Several TNF-α inhibitors have been shown to significantly improve the condition of patients with systemic sclerosis (37). TNF-α produced by T cells was involved in promoting the migration and differentiation of Ly6C^lo^ monocytes into pathogenic M2 macrophages, which may contribute to the activation of fibroblast and production of collagen, leading to tissue fibrosis (38). TNF-α released in the GI tract induced epithelial cell alterations and promoted the inflammatory reaction (39). In addition, TNF played a critical role in GVHD, as increased levels of TNF-α before HSCT was significantly correlated with severe GVHD. Several clinical studies have demonstrated that TNF-α blockade exerted promising activity in patients with GI-GVHD (40). In the current study, we demonstrated that IL-Y aggravated the progression of cGVHD by activating T and B cells, and increasing TNF-α secretion by CD4^+^ and CD8^+^ T cells in scleroderma-like and lupus-like cGVHD models. This is inconsistent with previous studies that IL-Y can exert an immunosuppressive effect by inhibiting the differentiation of Th1 and Th17 cells (21). It may be due to the different function of IL-Y in different animal models. Treg cells are critical mediators of immune tolerance and are required to prevent fatal autoimmunity in healthy individuals. Treg cell impairment is associated with loss of tolerance, autoimmunity and cGVHD (20, 41). In preclinical models of allo-HSCT, adoptive transfer of Treg cells can ameliorate GVHD without impairing therapeutic GVL responses (42). Impaired Treg cells reconstitution appears predictive for subsequent cGVHD studies (43). Indeed, we observed a significant down-regulation of splenic Treg cells in MC IL-Y group. Therefore, treatment strategies attempting to enhance Treg numbers by blocking the signaling pathway of IL-Y are attractive for cGVHD therapy, offering the possibility of therapeutic immune modulation without generalized immunosuppression.

Stimulation of CD4^+^ T cells and their interactions with autoantibody producing B cells have been proved to play critical roles in the pathology of cGVHD (9). Accordingly, 8 weeks after establishment of cGVHD, we observed that the frequencies of donor derived activated CD4^+^ T cells and ICOS^+^ Tfh cells were obviously increased in spleens of the recipients treated with IL-Y. Tfh cells are necessary for GC B cell formation and maintenance, which were shown to be required for the pathogenesis of cGVHD (44). Additionally, expression of ICOS was demonstrated to play critical roles in Tfh cells to mediate GC B cell reactions (45). In our study, although Tfh cells were not significantly increased, percentage of ICOS^+^ Tfh cells was significantly upregulated in the recipients given MC IL-Y plasmids, which was consistent with recent clinical reports (16). Furthermore, we found that IL-Y administration promoted the differentiation of GC B cells in recipient spleens. More importantly, higher levels of MHC-II and CD86 were expressed on donor B cells in recipient mice with MC IL-Y plasmids treatment, suggesting that IL-Y may affect B cell activation and antigen-presenting function through regulating T cell activation and differentiation.

IL-Y is a novel cytokine found to be involved in cGVHD pathogenesis in murine model of cGVHD. We have demonstrated that IL-Y could aggravate cGVHD and play pleiotropic roles in regulating the differentiation and function of multiple immune cells involved in the pathogenesis of cGVHD. IL-Y may selectively promoted TNF-α production by CD4^+^ and CD8^+^ T cells through IL-27R*α*, leading to the progression of cGVHD. Further studies are needed to reveal whether p40 (a component of IL-Y) could be involved in the diagnosis or prognosis of patients with allo-HCT who developed cGVHD. Taken together, our results provide evidence that targeting p40 (46) and IL-27R*α* signaling can be effective therapeutic strategies for cGVHD treatment.

## Data Availability Statement

The raw data supporting the conclusions of this article will be made available by the authors, without undue reservation.

## Ethics Statement

The animal study was reviewed and approved by Jiangsu Institute of Hematology, The First Affiliated Hospital of Soochow University, Institute of Blood and Marrow Transplantation, Collaborative Innovation Center of Hematology, Soochow University, Suzhou, China.

## Author Contributions

HL, DW, and YuL designed the study. LW, ZJ, and BH performed the research. KL, LL YoL, YS, YZ, HG, MX, YD, and YX contributed to the experiments. LW and ZJ analyzed the data. LW, ZJ, and YuL wrote the manuscript. All authors contributed to the article and approved the submitted version.

## Funding

This study was supported by the great form the National Key R&D Program of China (2017YFA0104502, 2017YFA0104500), National Nature Science Foundation of China (Grant No. 81500146, 81730003), National Science and Technology Major Project (2017ZX09304021), Jiangsu Social Development Program (BE2018651), Jiangsu Natural Science Foundation (BK20150356), Jiangsu Provincial Key Medical Center (YXZXA2016002), Jiangsu Medical Outstanding Talents Project (JCRCA2016002), Priority Academic Program Development of Jiangsu Higher Education Institutions (PAPD), Translational Research Grant of NCRCH (2020WSC05), Suzhou Science and Technology Development Project (SYS2019021).

## Conflict of Interest

The authors declare that the research was conducted in the absence of any commercial or financial relationships that could be construed as a potential conflict of interest.
