# Supplementary figures and images for: IL-Y Aggravates Murine Chronic Graft-Versus-Host Disease by Enhancing T and B Cell Responses
Source: Front Immunol. 2020 Nov 23;11:559740. doi: 10.3389/fimmu.2020.559740 (PMC7719702; doi:10.3389/fimmu.2020.559740)

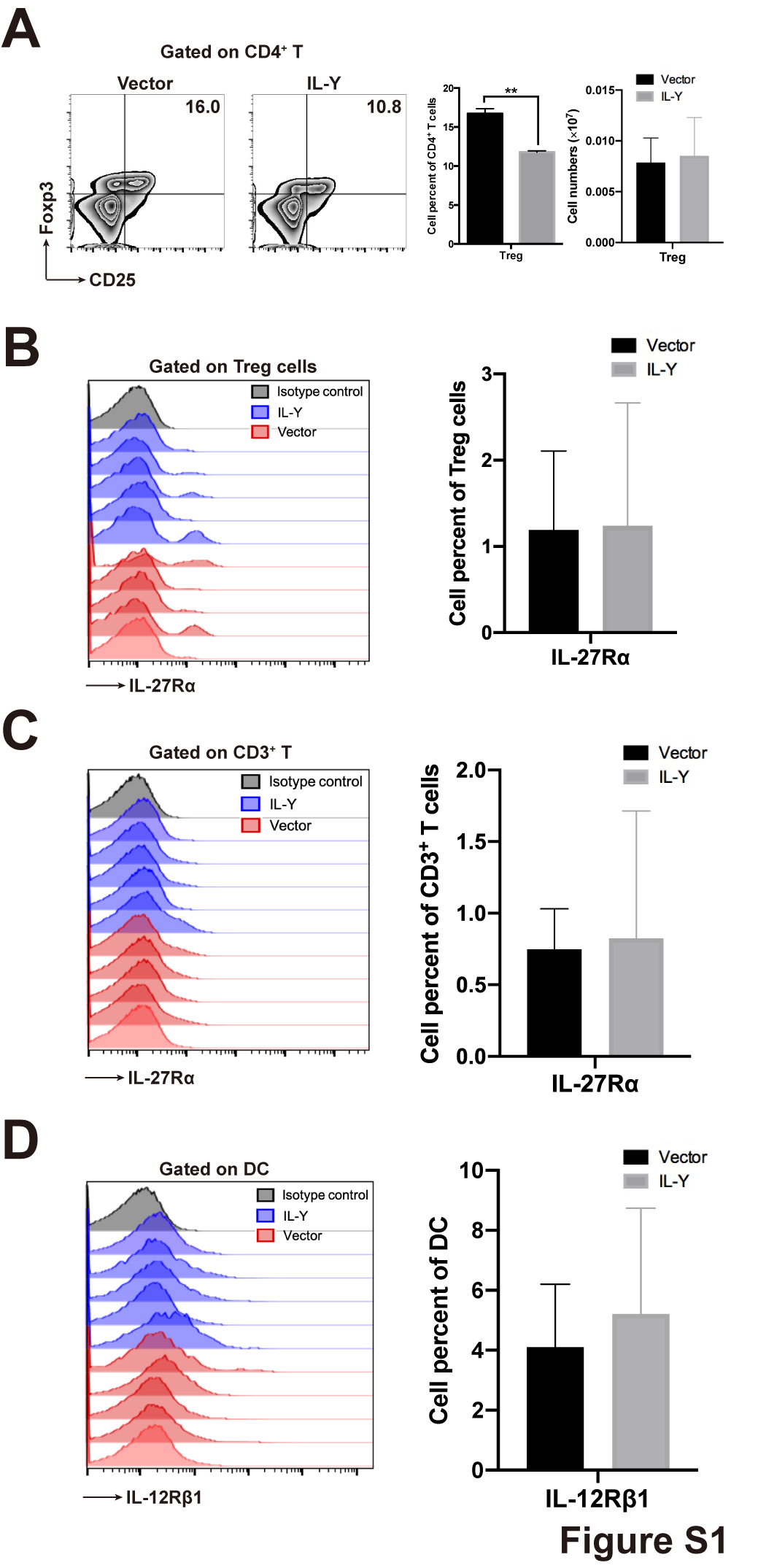

Supplement: Supplementary file 1 [file Image_1.tif]
